# Adeno-Associated Virus (AAV) Gene Delivery: Dissecting Molecular Interactions upon Cell Entry

**DOI:** 10.3390/v13071336

**Published:** 2021-07-10

**Authors:** Edward E. Large, Mark A. Silveria, Grant M. Zane, Onellah Weerakoon, Michael S. Chapman

**Affiliations:** Department of Biochemistry, University of Missouri, Columbia, MO 65201, USA; largee@missouri.edu (E.E.L.); msilveria@mail.missouri.edu (M.A.S.); zaneg@missouri.edu (G.M.Z.); opw4m7@mail.missouri.edu (O.W.)

**Keywords:** adeno-associated virus, AAV, gene therapy, virus structure, AAV receptor, AAV attachment factor

## Abstract

Human gene therapy has advanced from twentieth-century conception to twenty-first-century reality. The recombinant Adeno-Associated Virus (rAAV) is a major gene therapy vector. Research continues to improve rAAV safety and efficacy using a variety of AAV capsid modification strategies. Significant factors influencing rAAV transduction efficiency include neutralizing antibodies, attachment factor interactions and receptor binding. Advances in understanding the molecular interactions during rAAV cell entry combined with improved capsid modulation strategies will help guide the design and engineering of safer and more efficient rAAV gene therapy vectors.

## 1. Introduction

### 1.1. AAV Biotechnology

AAV is the basis of a multi-billion dollar industry and hundreds of clinical trials used AAV delivery systems [1]. Viral vector biotechnology is a leading choice for gene therapy platforms and recombinant Adeno-Associated Virus (rAAV) vectors are typically preferred due to low toxicity [2], dependence upon other viruses for replication [3], broad tropism and the ability to infect both dividing and non-dividing cells. There are currently two AAV gene replacement treatments available for autosomal recessive genetic disorders: Luxturna and Zolgensma. Luxturna rAAV is based on the AAV2 serotype (rAAV2) and delivers a functional copy of the RPE65 (retinal pigment epithelium-specific 65 kDa protein) gene to the retinal pigment epithelial cells of patients with retinal dystrophy [4]. Zolgensma uses rAAV9 to deliver a functional copy of the human SMN1 (survival of motor neuron 1) gene to spinal muscular atrophy (SMA) patients [5]. SMA is the most common fatal single gene disease in infants, and it is caused by an autosomal recessive mutation in the survival motor neuron 1 gene (SMN1). More rAAV therapies are in the pipeline, and dozens of clinical trials are currently underway [6]. Thus, the range of rAAV treatment options and efficacy of AAV as a therapeutic vector continues to grow.

rAAV is a successful gene therapy biopharmaceutical, but more efficient rAAV vectors that can be delivered at lower doses are still needed. For example, high rAAV doses were implicated in the recent tragic deaths of three AAV gene therapy patients during X-linked myotubular myopathy Phase I/II clinical trials [7]. The trial administered an AAV8 serotype containing a wild-type copy of the MTM1 gene at various doses. Immune toxicity was observed in those with liver disease, obesity or at older age at the highest dose (3 × 10^14^ vg/kg; vector genomes/kilogram) [7]. The immunotoxicity of rAAV is less severe than adenoviral or lentiviral vectors [8,9], but these patient deaths are a grim reminder of the need for improved AAV vectors that can be delivered at lower doses. rAAV vectors that avoid antibody neutralization or vectors with improved transduction efficiency would significantly reduce the rAAV gene therapy doses needed to cure previously incurable diseases.

rAAV production continues to be updated and evaluated for industrial-scale production of next-generation rAAV therapies. Commercial and research rAAV production typically involves a “triple transfection” approach. Triple transfection components consist of a plasmid encoding the transgene, a helper plasmid containing Adenovirus type-5 (Ad5) helper genes (i.e., E1a/b, E2a, E4 and VA RNA) or their equivalents and another plasmid encoding the rAAV Rep and Cap proteins [10,11,12,13]. Variations include combined helper and rAAV plasmid functions on a single plasmid or stable expression of helper functions, pre-programmed into mammalian cell lines [14]. Vector production improvement focuses on particle metrics such as the ratio of empty to non-empty vector particles. Increasing the number of full particles decreases the number of particles needed for gene therapy and subsequently improves safety and efficacy [15].

rAAV gene therapy technology is derived from a small (~25 nm diameter) non-enveloped wild-type AAV (wtAAV) ssDNA virus in the family *Parvoviridae*. wtAAV serotypes from diverse primate lineages must navigate common human biological barriers. Human wtAAV serotypes have been studied extensively, and the most well-understood primate family members are serotypes AAV1-9. Serological and sequence classifications indicate unique origins for the most divergent serotypes, AAV4 and AAV5. Other serotypes can be grouped by clade, with representative serotypes listed parenthetically: A (AAV1/6), B (AAV2), C (AAV3/13), D (AAV7), E (AAV8/10) and F (AAV9) (Table 1) [16]. Four additional serotypes (AAV10-13) [17,18,19] were isolated after the classification of AAV1-9. Structural similarities of AAV11 and AAV12 to AAV4 place the three in a potential clade whereas structural similarities of AAV13 to AAV3 suggest AAV13 is in clade C [20]. These primate serotypes serve as the primary basis for rAAV biotechnology.

rAAV capsids face several significant hurdles before uncoating in the nucleus, and this is an essential area of research for improving vector designs. First, the capsids must avoid pre-existing antibodies and interact with cellular attachment factors and cell entry receptors. After cell entry, rAAV vectors are trafficked to the nucleus where rAAV particles are uncoated. This review focuses on rAAV vector engineering strategies and known cell entry interactions affecting rAAV transduction. It will focus on the capsid, recognizing that, in terms of immune response, the transgene can have varied specific effects, and that vector DNA or derived RNA could also trigger immune responses, topics that are garnering increasing attention and have been reviewed recently elsewhere [35].

### 1.2. AAV Molecular Biology

AAV2 is the canonical AAV serotype (or “type species”) for the AAV family and provides a valuable reference for studying other AAVs. For example, AAV2 was used to characterize the first AAV glycan attachment factors and deduce the first high-resolution AAV structure, and AAV2 served as the basis for the first genome-wide screen to identify protein entry receptors [22,24,36,37]. More recently, AAV2 was the model serotype for systematic genotype-phenotype investigation [38].

**Figure 1:**
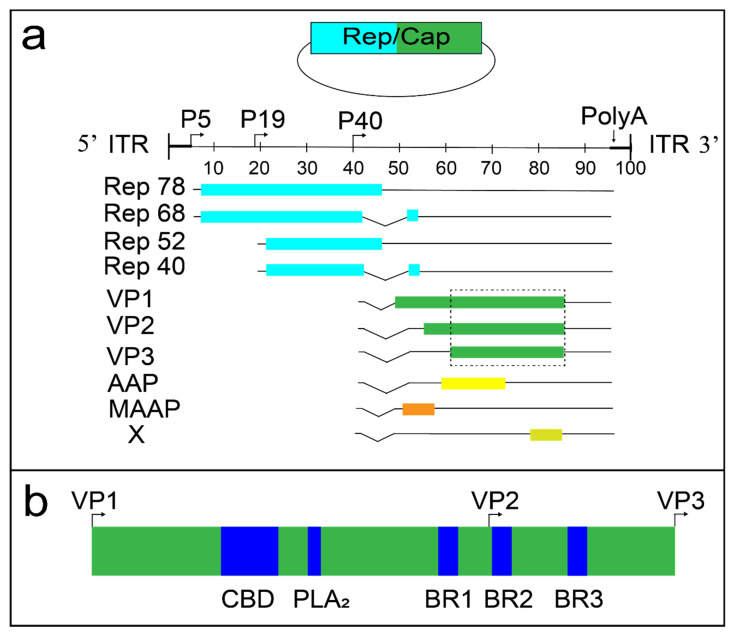
The AAV2 genome and VP1/2 region. (**a**) Rep/Cap plasmids (top of panel) encode multiple proteins (light blue = Rep; green = Cap). The linear ssDNA wtAAV2 genome is flanked by inverted terminal repeats (ITRs). Four Rep proteins are produced in the 5′ coding region (left side = light blue). VP1-3, AAP, MAAP, and X proteins are expressed in the 3′ region (right side = green). The dashed line rectangle surrounds the VP3 common region of VP1-3, which produces known native AAV structures (Table 1). (**b**) The VP1/2 region contains basic regions 1-3 (BR1-3), a phospholipase A_2_ (PLA_2_) enzyme and a calcium binding domain (CBD).

The AAV2 genome contains ~4.7 kb of ssDNA flanked by inverted terminal repeats (ITRs) and encodes two primary gene sets; *rep* and *cap* (Figure 1a). The 5′ region of the genome encodes four Rep proteins from a single open reading frame (ORF) (Rep40, Rep52, Rep68, and Rep78). The Rep proteins are involved in virus replication, packaging and integration. Rep78/68 possess helicase/endonuclease activity and bind to the ITRs [39], while Rep52/40 possess 3′-to-5′ helicase activity and are responsible for the packaging of viral genomes into the capsid [40]. The 3′ genome region encodes the three AAV capsid proteins VP1, VP2, and VP3 in the same ORF with respective sizes of 87 kDa, 72 kDa, and 62 kDa. The AAV2 VP3 region (533 amino acids (aa)) is common to VP1-3, and structures of the N-terminal regions unique to VP2 (65 aa) and VP1 (202 aa) remain unsolved (Figure 1b). The unique region of VP1 (abbreviated VP1u) contains a phospholipase domain important in AAV trafficking from endosomes to the nucleus [41]. The VP1-3 genomic region encodes the proteins that form the viral capsid and, in turn, determines both antibody recognition epitopes and receptor binding sites.

In addition to VP1-3, the ~2.2 kb *cap* region encodes at least three known alternate reading frames (i.e., overprinted regions; Figure 1a). The 5′ region of the AAV2 *cap* coding region contains the recently discovered 119 amino acid MAAP protein [38] and the AAP protein (204 aa) [42,43,44,45,46,47]. The X gene encodes a protein of 172 amino acids at the 3′ end of the VP1 coding region [38,48,49,50]. The functions of these overprinted regions continue to be an active area of research.

The VP1u domain is important for rAAV transduction. The first three of four basic regions (BR1-4) are involved in nuclear localization [51] and BR1 is specific to VP1u while BR2-3 are part of VP1/2. The AAV VP1u domain also encodes a calcium-dependent group XIII phospholipase A_2_ (PLA_2_) enzyme [52] and a calcium-binding domain. The enzymatic activity of PLA_2_ is vital for AAV endosome escape [41,53]. Multiple structures exist for other PLA_2_ enzymes in unbound or inhibitor-complex states, but no structures exist for parvovirus versions.

The structural properties of the AAV capsid are crucial determinants in rAAV production, purification, and cargo delivery. Cap proteins must form a stable 60-mer capsid containing the desired transgene cargo. The amino acid sequence of the *cap* region of rAAV plasmids also encodes capsid regions responsible for tissue tropism, antigenicity and interactions with cell entry factors [54]. Given the role of the capsid in delivering therapeutic transgenes into the cell and trafficking to the nucleus, capsid structure will be a key focus of this review, both as a foundation for understanding and modulating the underlying molecular interactions and as a foundation for understanding the limits on modifying the virus in immune evasion.

### 1.3. The First High-Resolution Dependovirus Structure

Among the first atomic structures for icosahedral capsids were plant viruses with a conserved β-barrel motif [55]. The motif consists of eight antiparallel β strands [56] and is also known as a jelly-roll [57]. The β-barrel is not fully continuous but is more aptly described as a sandwich of two sheets, each comprised of at least four antiparallel strands (lettered BIDG and CHEF). As in many other viruses, the jelly-roll β barrel is the foundation for parvovirus capsid proteins, but some of the loops between strands are several times longer than in those previously seen [58].

**Figure 2:**
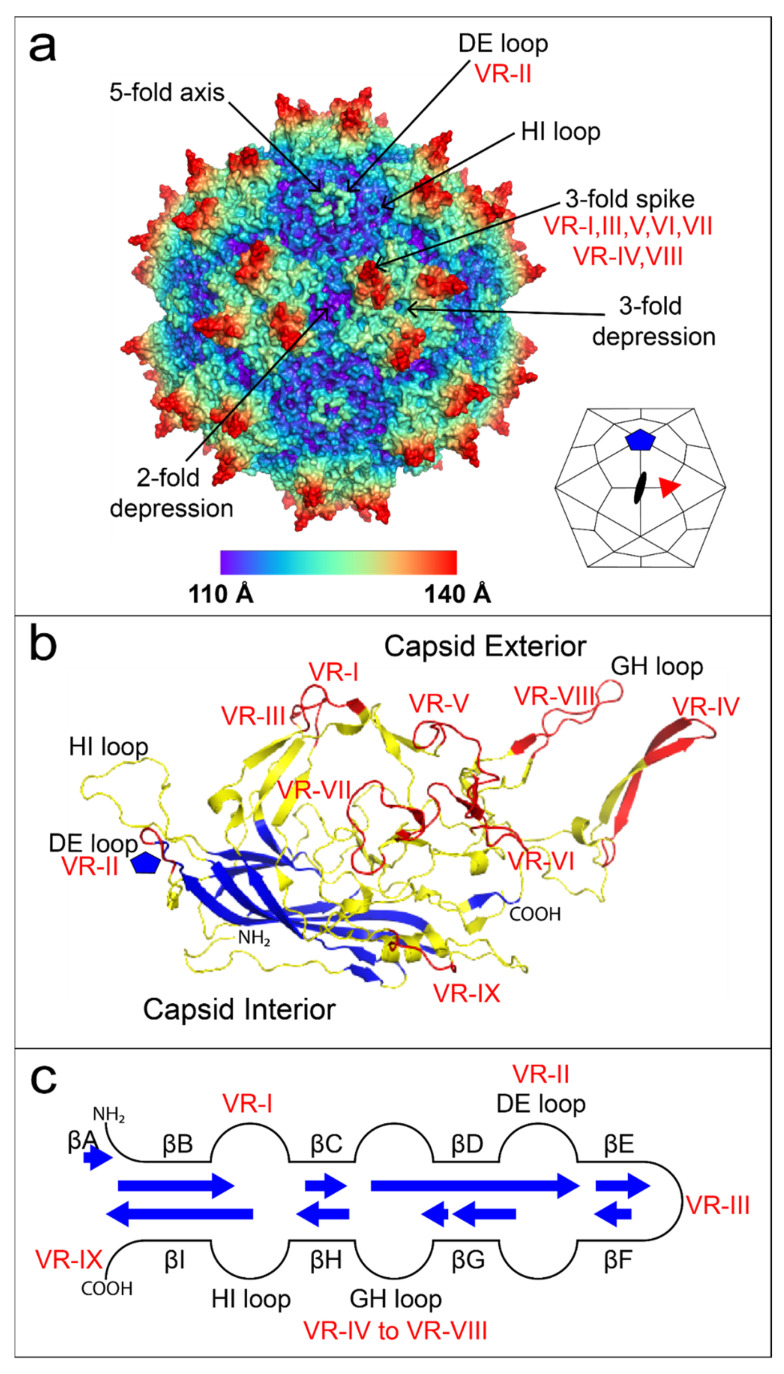
The AAV2 structure. (**a**) AAV2 60-mer colored by radial distance from the center of the virion; 2-fold, 3-fold, and 5-fold axes are indicated by arrows and associated variable regions (VR) are noted. A small icosahedron is provided in the lower right corner showing 2-fold (black oval), 3-fold (red triangle), and 5-fold (blue pentagon) axes along with kite-shaped quadrilaterals to denote schematically the approximate juxtaposition of subunits. (**b**) AAV2 jelly-roll β barrel strands form the inner surface of the capsid subunit and are highlighted in blue. AAV loops constituting more than half of the structure, colored yellow, are on the outer surface of the capsid. Sequence-variable regions are highlighted in red. (**c**) The topology of the β barrel is shown schematically in the center, with blue arrows of length proportional to the number of strand residues. Loops vary in size and complexity. Structures in panels (**a**,**b**) were prepared using PyMOL [66].

Parvovirus capsids comprise sixty monomers in icosahedral symmetry (T = 1). The family contains three subfamilies: *Parvovirinae*, *Densovirinae,* and *Hamaparvovirinae* [59]. *Parvovirinae* infect the dividing cells of vertebrates and *Densovirinae* infect dividing arthropod cells while the newly classified *Hamaparvovirinae* (Greek *hama* = together) subfamily contains members that infect vertebrate and invertebrate hosts [59]. Four high-resolution *Parvoviridae* family structures provided early insights into autonomous vertebrate *Parvovirinae* structures: canine parvovirus (CPV; Tsao et al., 1991 [58]), feline panleukopenia virus (FPV; Agbandje et al., 1993 [60]), minute virus of mouse (MVM; Agbandje-McKenna et al., 1998 [61]), and porcine parvovirus (PPV; Simpson et al., 2002 [62]). Key features of parvovirus structures include a 3-fold proximal spike which varies in prominence, a large shallow canyon surrounding the 5-fold axis, and a small depression/valley centered on the 2-fold axis between two 3-fold spikes (Figure 2a). An invertebrate parvovirus structure from the *Densovirinae* subfamily, the wax moth (*Galleria mellonella*) densovirus (*Gm*DNV), indicated significant differences from the vertebrate parvovirus structures [63]. The β barrel topology is conserved but the long GH loop of vertebrate parvoviruses is mostly missing [57,64]. The absence of a long GH loop in *Gm*DNV leads to a much flatter surface topology than for the other parvoviruses that face adaptive immune systems.

The 3 Å X-ray crystallography structure of AAV2 [22] was the first high-resolution *Dependovirus* structure and provided structurally-guided insights into virus capsid evolution (Figure 2a). AAV and autonomous parvovirus VP1 amino acids sequences are not similar (<24% amino acid identity) [65]. Nevertheless, there is high structural homology with the mammalian autonomous parvoviruses, sharing the β barrel and the presence of a long GH loop, if not details of its structure [22,57]. AAV serotype structures have variable regions (VR) with higher sequence diversity [24]. The majority of VR are found in the GH loop within regions responsible for antibody evasion and cell entry (Figure 2b,c). Therefore, the AAV2 structure provided a much-needed reference for understanding *Dependovirus* diversity and a three-dimensional map for understanding the molecular mechanisms of cellular entry.

## 2. AAV Capsid Modification Strategies

### 2.1. Natural AAV Genetic Diversity

Naturally occurring AAV capsid amino acid diversity is a useful resource for variation of functional properties. Over 100 serotypes have been isolated from humans and non-human primates [16,67]. AAV capsid properties modulate tissue tropism (Table 1) and capsid diversity may improve immune evasion and tissue tropism properties.

Bats are a rich source of AAV genetic diversity [68,69] and may provide capsids with improved tropism and immune evasion properties. For example, bat AAV sequences have low capsid sequence identity (<60%), reduced antibody neutralization profiles, and increased muscle to liver transduction ratios compared to primate AAVs [70]. Bat AAV10HB was chosen for additional structural studies, and divergent loop structures were observed between AAV2, AAV5, and AAV10HB. The β-barrel motifs of AAV2, AAV5, and AAV10HB are highly similar. The root-mean-square deviation (RMSD) between the C_α_ atoms of VP3 within the conserved β-barrel strands and α-helical region of AAV2, AAV5, and AAV10HB is ~0.5 Å. The local RMSD of the VR-II, VR-VIII, VR-IX, and HI loops is less than 2 Å whereas all other VR loops have a local RMSD greater than 2 Å. Neither A20 (AAV2-specific) or ADK5b (AAV5-specific) antibodies recognize 10HB [29]. Bat AAV10HB is the first and only non-primate AAV structure [29]. More comparisons structure–function are needed to deduce capsid residues responsible for antibody evasion. Bat AAVs can be used to illustrate potential strategies of improving vector immune evasion: (1) vectors based on capsids of AAVs from non-human hosts to which humans would be immune-naive; (2) rational engineering of chimera, replacing surface loops containing known human neutralizing immunogenic (NIm) sites with corresponding loops from non-human AAVs that are not cross-neutralized; (3) incorporation of the same non-human AAVs in the pool used for AAVDJ-like selection of new variant vectors [34]; or site-directed escape mutation of NIm sites as they are identified within capsid structures. Each of these capsid-modification strategies is further explored within the section.

wtAAV is constantly evolving and novel variants can be isolated from original cultures or after virus propagation. For example, AAV3 is closely related to AAV2 and both serotypes were isolated from humans [71]. A comparison of AAV2 and AAV3 DNA sequences revealed that they were distinct but closely related serotypes [72]. AAV3 was re-isolated from the original AAV3 wild-type virus stock and an additional isolate (AAV3B) was discovered. Sequencing revealed a difference of six amino acids [72,73]. Virus evolution during laboratory passaging might have been even more consequential for AAV2. Human AAV2 isolates typically do not attach to HSPG until after adaptation to cell culture [74,75]. More recent studies indicate AAV2 adaptation quickly yields subtypes with reduced liver tropism and increased glycan attachment or increased liver tropism and reduced glycan attachment [76]. Finally, advances in high throughput long-read sequencing provide opportunities to discover AAV capsids (e.g., AAVv66 from AAV2) from human tissues with unique tropism and antibody evasion properties [77]. Therefore, some natural AAV capsid diversity is potentially present in wild isolates, and AAV capsid differences can quickly emerge during laboratory propagation.

Paleovirology and AAV ancestral reconstruction approaches provide another means to harness natural AAV diversity. Viral genome integration occasionally occurs in animal germ cells, and these ancient insertion events are ubiquitous in extant animal genomes. The insertion events are referred to as “EVEs” (EVEs: Endogenous Viral Elements), and ancient AAV insertions are AAV-EVEs [78]. Comparisons of AAV-EVEs from sequenced mammalian host genomes provide estimates of divergence times based on the last common ancestors of EVE hosts. Current estimates suggest AAV integrated into mammalian genomes 23–77 millions of years ago (MYA) [78].

Capsid differences are observed between extant primate AAVs and ancient mammalian AAV-EVEs [78]. Some of the differences are found in the mammalian AAV-EVE VP1u domain. The AAV-EVE PLA_2_ domains have similar loss of function mutations but have intact calcium-binding sites [78]. Mutant PLA_2_ paired with functional calcium-binding sites indicates a potential selective advantage for calcium-dependent functions with the concurrent absence or reduction of PLA_2_ activity.

Exploration of intermediates between EVEs and AAVs can be further evaluated using ancestral sequence reconstruction (ASR) by phylogenetic analysis [79]. AAV ASR uses known AAV capsids to generate predicted ancestral capsid sequences with potentially beneficial attributes [80,81]. The primate AAV serotypes 1–3 and 7–9 are the primary focus of many clinical studies, and ASR identified a recent primate AAV ancestor, Anc80L65, as a novel vector with potentially valuable properties [80]. A phylogenetic tree was constructed using 75 primate AAV sequences along with predicted ancestors at each branch node. Based on its phylogenetic position, the Anc80 ancestor was chosen to create an Anc80 library (Anc80Lib) consisting of 776 clones representing the predicted amino acid variability at the Anc80 node. One clone, Anc80L65, was chosen for additional studies and showed increased transduction properties and reduced antibody neutralization. The utility of this approach is highlighted by the recent use of rAnc80L65 to restore balance and hearing loss in neonatal and embryonic mouse models [82,83].

Interesting differences are also observed in the capsid residues of ASR AAV tree nodes. A homology model of the Anc80 node structure was created using the AAV8 crystal structure (PDB: 2QA0) [80]. The majority of predicted structural differences between AAV2, AAV8, and Anc80 are located near the 3-fold spikes [80]. Residue differences between Anc80, predicted AAV2 ancestral nodes, and AAV2 were superimposed on predicted T cell and antibody epitopes [84,85]. Capsid differences in the lineage leading to AAV2 tend to aggregate near predicted epitopes [80]. This evidence suggests a virus evolutionary model in which immune recognition significantly influences the evolutionary trajectory of primate AAVs.

Beyond sequence–structure variation within capsid subunits, another variable that can be modulated is the ratio of VP1-3. VP1 is important for cell transduction [86,87]. Wild-type AAV2 has a VP1-3 ratio of 1:1:10 [88,89], equating to about 3–6 VP1 monomers per capsid [90]. A modest decrease in VP1 levels is detrimental to cell transduction while an increase in VP1 levels and decrease in VP2 levels (VP1:VP2:VP3 equals 1.9:0.1:8) increases transduction [91]. Note that while overexpression of VP1 increases transduction, it also leads to reductions in rAAV yield [91]. The underlying mechanism is unknown, but reduced rAAV production might be associated with VP1u-associated protease activity and subsequent self-digestion or insufficient levels of VP2/3 for AAV packaging [91,92]. As for recombinant expression systems, one baculovirus system generates reduced levels of VP1 [93], but modifications can increase VP1 to wild-type (VP1 equals 10% of VP3) levels [94]. The published VP1 maximum is a VP1-3 ratio of 1.9:0.1:8 [91]. Optimized levels of VP1 may further improve rAAV transduction efficiency.

AAV vector “pseudotyping” is a well-established technique in which the genome of one virus is encased by a different serotype, a different virus or a synthetic virus [95]. AAV capsid monomers from different plasmid sources can also form “mosaic” rAAV particles with altered transduction efficiency. Mosaic rAAV are typically avoided for current gene therapy approaches [96], but researchers continue to identify mosaic AAV capsids with unique biochemical and transduction properties [97,98,99,100,101].

### 2.2. AAV Directed Evolution

**Figure 3:**
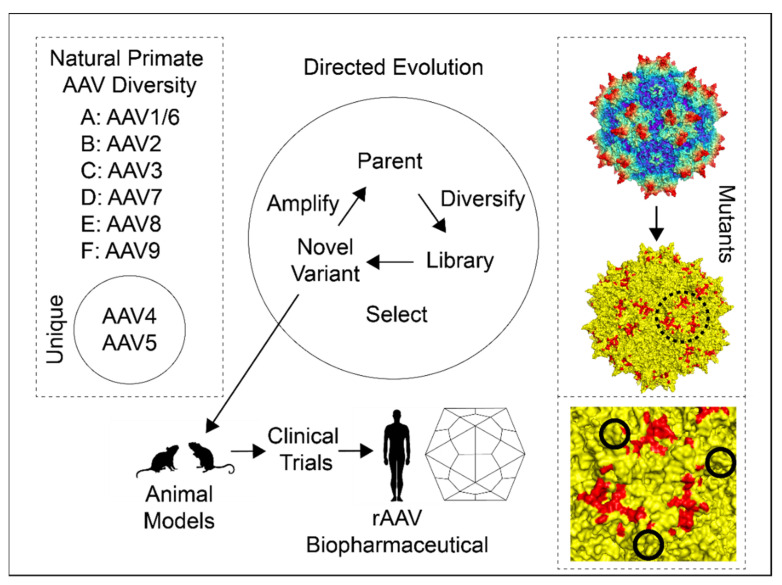
Capsid modification strategies. The three major approaches to capsid modifications are, from left to right: natural diversity (left dashed line box), directed evolution (middle), and mutants (right dashed line box). Natural primate AAV diversity includes the major and unique AAV clades. Directed evolution begins with parental serotypes, and these are diversified via recombination-based techniques (see text for details). This creates an AAV library that can be screened with selection of novel variants. Novel variants can then be amplified for additional rounds of selection or tested in animal models as a candidate rAAV biopharmaceutical. The mutants box consists of an AAV2 60-mer corresponding to Figure 2a at the top and a mutated yellow AAV2 60-mer at the bottom with mutations highlighted in red. The dashed-line circle surrounds the 3-fold axis, and a close-up view of this region is shown in the bottom right dashed box. Solid line circles show the peaks of each axis and the 3-fold depression is in the center. The three ~3.5 nm diameter red patches are “dead zones” first identified in Lochrie et al., 2006 [118]. Comparison of the top and middle models shows that the dead zone is mostly on a plateau between spikes where the PKD2 domain is bound in the AAV2-AAVR complex [119,120]. A summary of these mutations and others is provided in Supplementary Table S1. Structures were prepared using PyMOL [66].

Another AAV capsid modification strategy uses the power of directed evolution (Figure 3). Directed evolution uses artificial selection to engineer changed molecular phenotypes. RNA was the first target molecule of directed evolution [102] but this process has since then been applied to proteins [103,104,105] and protein enzymes [106]. The importance of directed evolution was recently recognized with the 2018 Nobel Prize in Chemistry [107].

rAAV capsids encode both enzymatic (e.g., VP1u PLA_2_) and structural properties vital for cell entry, and these properties can be improved via directed evolution. Directed evolution of protein sequences typically relies on “DNA shuffling” of the DNA encoding the protein [108,109]. DNA shuffling utilizes the power of recombination between similar DNA sequences (i.e., homologous recombination) and can utilize fragmented DNA and/or PCR-based reassembly. Homologous recombination occurs naturally in parvovirus [110] and wtAAV populations [16] and is also observed in laboratory cultures of AAV-infected cells [111]. Following recombination, clonal isolates of AAV recombinants can be identified for further characterization [112].

Directed evolution of AAV capsids relies on large diverse AAV capsid DNA libraries to select candidate AAVs. Libraries are built using homologous recombination-based shuffling of selected parental AAV DNA. Error-prone PCR, with or without a staggered extension process, can be used to increase library genetic diversity [113,114,115]. Recombination can be further enhanced using capsid DNA sequences from closely related serotypes or codon-optimized capsids [116]. Optimized directed evolution pipelines generate DNA libraries with sufficient genetic diversity to select for infectious isolates [117]. Subpopulations can then be isolated using additional selection steps [117]. Selection schemes can be applied in vitro and/or in vivo to clone chimeric AAVs with favorable tropism, reduced immune neutralization, or desirable biochemical properties.

A significant advance in tracking and evaluating large complex AAV capsid DNA libraries is the use of DNA barcodes (i.e., AAV barcode-Seq) [121]. DNA barcodes can be used to measure the fitness of mutations over time [122]. The combination of DNA barcodes and high-throughput sequencing provides additional means to identify variants from scanning libraries with favorable traits, such as tropism to particular tissues, in vivo [121,123,124]. Subsequent research has validated the use of barcodes as a standard approach to managing and tracking a wide range of AAV libraries [38,45,123,124,125,126,127,128,129].

Directed evolution approaches have yielded a number of chimeric AAV constructs that were subsequently developed as rAAV vectors, often with the goal of modulated tropism [34,130,131,132]. AAV-DJ is the most fully characterized and is a chimera of AAV2, AAV8, and AAV9 derived from the DNA shuffling of eight different AAV serotypes targeting human liver [34]. Hybrids of eight parental serotypes (AAV2, 4, 5, 8, 9, avian (bird) AAV, bovine (cow) AAV, and caprine (goat) AAV) were initially passaged through human liver cells leading to a more select population of hybrids in which only five serotypes were represented (2, 4, 5, 8, and 9). Following liver transduction, the population was further reduced to a single hybrid of three serotypes (2, 8, and 9) by selection for resistance to pooled neutralizing human antisera (intravenous immunoglobulin or IVIg) [34]. The AAV-DJ IVIg-escape variant differs from its closest single parent (AAV2) at 60/737 amino acids (~8.1%) [34]. AAV-DJ was the most efficient serotype for in vitro transduction of 15 cell lines from multiple tissues and species [34]. AAV8 and AAV9 are superior to AAV2 for liver transduction, and AAV-DJ was on par with AAV8/9 in vivo liver expression [34]. Gene therapy may require multiple doses, and an immune response typically neutralizes reinfusion of the primary vector. Prior use of AAV-DJ did not elicit a neutralizing immune response that was able to block subsequent transduction with rAAV2, rAAV8, or rAAV9. The most striking difference between AAV2 and AAV-DJ is observed at VR-I, which corresponds to the epitope of neutralizing mouse monoclonal antibody (mAb) A20 [30,133,134,135]. Therefore, AAV-DJ is a product of directed evolution with superior transduction and antibody evasion properties compared to its closest related ancestor (AAV2). It seems likely that rigorous selection for IVIg-escape generated change at a known neutralizing epitope, and this also affected cellular tropism [30].

A possible cautionary tale of the power of selection was the recent development of an AAV variant that can cross the blood–brain barrier (BBB). AAV9 had early promise as a treatment for CNS disorders because strong CNS expression was observed in small and large mammalian models [136,137,138] and AAV9 can pass through the mouse BBB but to a limited extent [136]. A novel Cre recombination-based AAV targeted evolution (CREATE) approach was developed to improve CNS transduction efficiency, and an AAV-PHP.B variant was identified [139]. CREATE utilizes libraries consisting of heptapeptide insertions into a permissive AAV9 site that corresponds with AAV2 glycan attachment (amino acids 588/589; Section 3.1). Intravenous administration of AAV-PHP.B showed promising transduction (~50-fold improvement) in the entire adult mouse CNS and abrogated the non-CNS expression that would have been expected of AAV9. Thus, the improved transduction efficiency of AAV-PHP.B in the CNS of a mouse model provided a promising lead for human CNS rAAV gene therapy.

A follow-up study showed, however, that AAV-PHP.B was specific to the C57BL/6J mice used for in vivo selection [140] and not BALB/cJ mice. This observation quelled excitement over the potential translational use of AAV-PHP.B but provided the basis for mapping the LY6A genetic variants responsible for unlocking the C57BL/6J BBB [141]. LY6A is a GPI-anchored protein, and GPI-protein-mediated transport has been suggested as a determinant of AAV2 and AAV5 transduction [142,143]. Consequently, one could postulate that LY6A is an attachment factor or receptor that enables transport through the BBB. Meanwhile, another AAV9 variant (AAV-F) has been identified with superior CNS transduction properties using the iTransduce version of CREATE in the same permissive AAV9 site, and AAV-F does not utilize LY6A [144]. One lesson from AAV-PHP.B is that there is a danger of using (well-characterized) in-bred animal models to select viral vector traits that are only compatible with host genotypes not found in human populations. Another lesson is further encouragement to modulate tropism by selection from a library in which peptide sequence is randomized locally at a site that is surface accessible in the structure and available to interact with host factors [139,145].

### 2.3. AAV Mutants

Early AAV capsid analyses were directed towards mapping epitopes and glycan attachment sites using peptide scanning (PEPSCAN), peptide competition, and site-directed mutation of recognizable sequence motifs [134,146,147,148]. The atomic structure of AAV2 [22] opened the way for more rational mutation analyses. For example, the AAV2 glycan attachment site was now clearly visible and, of the basic amino acids with potential to interact with heparan sulfate [36], five were now seen on the capsid exterior (See Section 3.1.1). The AAV2 structure provided the necessary details to interpret previous mutagenic studies and to move forward with structurally-informed screens.

A particularly noteworthy study by Lochrie et al. investigated several phenotypes, following systematic mutation that screened a wide area of the accessible outer surface of AAV2 [118]. Of 145 amino acids on the outside surface, 64 of the most exposed (~55% of surface area) were targeted as potential antibody-binding epitopes. The screen validated predicted basic amino acid residues responsible for heparin attachment. An additional circular area composed of eighteen amino acids (7 acidic and 1 basic) was also identified. Mutations in this ~3.5 nm diameter “dead zone” (Figure 3) decreased in vitro transduction independent of heparin-binding [118].

Antibody neutralization was also investigated. The mouse A20 antibody is well-known for AAV2 neutralizing activity, and a cluster was identified with five adjacent residues and one nearby as sites of neutralization of escape mutants. These mutants did not affect heparin attachment or transduction activity [118]. Similarly, three potential epitopes consisting of mutations that evaded three different samples of human sera from factor IX-deficient hemophiliacs were identified. One of these sites overlapped with the A20 epitope [118]. Finally, Lochrie et al. used human IgG (IVIg), pooled from >1000 individuals, to scan for additional epitopes. The suite of mutations responsible for AAV2 Nab evasion provides targets for stealth rAAV gene therapy vectors.

One exciting new addition to the AAV genetic manipulation toolkit utilizes a systematic approach to investigating AAV [38]. The entire capsid genome of AAV2 was modified, barcoded, and subjected to thermostability and transduction assays. The study confirmed the earlier observation [118] that the 3-fold spike is more tolerant of mutations compared to the 5-fold axis. Furthermore, by correctly identifying amino acids at the previously determined binding site of neutralizing antibody A20 [135], proof of principle was achieved that the approach could be used to map interaction sites.

The ability to monitor large AAV libraries also allows for improvements in rAAV capsid engineering via the design and implementation of computational models. Machine learning (ML) methods improve protein modeling and engineering [149], and the rational design of AAV capsids also benefits from ML. For example, a ML model was used to engineer AAV2 variants with superior liver transduction compared to AAV2 variants selected from randomly mutagenized populations [38]. Viable capsid formation is the first step of vector production, and ML models of capsid viability provide a useful measure of fitness. ML-based AAV libraries improved diversity in the 3-fold spike [128] and found unexpected differences in selection pressure for residues affecting capsid stability [150]. Another study started with electron microscopy imaging of AAV-antibody complexes, using pseudo-atomic models of multiple anti-AAV8 antibodies to identify important AAV8 epitopes at ~12–25 Å resolution [151]. AAV8 target residues were subjected to saturation mutagenesis, and the library was screened using human liver cells. A variant with improved transduction profiles and increased immune evasion was isolated, evidencing the utility of this approach [151]. Large AAV libraries engineered using computational models are poised to produce rAAV vectors with improved rAAV capsid properties such as viability, tropism, thermostabilty, and antibody evasion.

## 3. AAV Cell Attachment and Entry

### 3.1. AAV Glycan Attachment Factors

#### 3.1.1. HSPG

Extracellular glycans (e.g., glycoproteins, glycolipids) are common virus attachment factors and heparan sulfate proteoglycan (HSPG) attachment is used by several AAV serotypes prior to cell entry [54]. Heparan sulfate (HS) is a glycosaminoglycan that is covalently attached to protein. HS and heparin are negatively charged linear polysaccharides with heparin being the more sulfonated of the two forms. The most common disaccharide in HS (~50%) is glucuronic acid, linked to N-acetylglucosamine. HS and heparin interact with positively charged amino acids such as arginine and lysine [36,152]. Interactions of HS and heparin with proteins predominantly occur via electrostatic interactions with minor contributions from hydrogen bonds, hydrophobic effects and van der Waal interactions [153,154].

With the discovery of AAV2 HS-attachment [36], questions remained regarding the importance of HSPG in AAV infection. The 3.0 Å structure of AAV2 [22] and mutagenic studies [155,156] revealed the location of the AAV2 HSPG attachment site clustered on the side of each 3-fold spike (Table 2). The site was narrowed down to five basic amino acid residues responsible for HSPG-attachment (R484, R487, K532, R585, and R588) with the most severe loss of HSPG attachment occurring via R585 and R588 mutations in three major mutagenic studies [118,155,156]. The 8.0 Å cryo-EM structure of AAV2 complexed with heparin [133] confirmed the predicted HSPG attachment site derived from the AAV2 crystal structure [22]. Some of the positively charged residues responsible for AAV2 HSPG attachment (R585 and R588) are not conserved in other serotypes [133], which necessitated additional studies.

Follow-up structures of related serotypes indicated that the location of the AAV2 HSPG attachment site is not conserved across serotypes. AAV6 also attaches to HSPG and the 3.5 Å X-ray crystallography structure of AAV6 revealed a divergent but overlapping HSPG attachment site confirmed via site-directed mutagenesis [26]. AAV6 is missing the positively charged AAV2 R585 and R588 residues but compensates with two positively charged lysines at a different location (K459 and K493). Preliminary evidence suggested the AAV2 and AAV6 sites were more similar to each other compared to AAV2 and AAV3B HSPG attachment sites [23]. AAV3B is closely related to AAV2 and both serotypes were isolated from humans [71]. AAV3B also attaches to HSPG [97,161], but AAV3B lacks the AAV2 residues with the strongest interactions: R585 and R588. Other AAV3B residues could be postulated to fulfil an analogous role (R447 and R594). AAV3B was complexed with the sucrose octasulfate (SOS) heparin analog to generate a 6.5 Å X-ray crystallographic structure with sufficient resolution to identify the binding site which was on a 3-fold axis, where three copies of R594 come together [157]. In the cases of AAV2 and AAV3B, it was shown, through chimeric mutation, that electrostatic potential contributed by different amino acids was additive in its effects upon heparin-binding, cell binding, and in vitro cell transduction [157]. Thus, while binding using analogous interactions, the sites on AAV2 and AAV3B do not correspond. Therefore, AAV HSPG attachment sites exhibit convergent evolution with selection of positively charged residues. 

Aware that at some point, conformational changes would be needed for the release of VP1u and DNA, one question was whether glycan-binding induced the presumed changes [162]. Such speculation was inspired by other viruses; for example, the attachment of HIV to heparin sulfate and subsequent conformational changes are critical components of HIV co-receptor binding [163]. The 8.3 Å cryo-EM structure of a 17 kDa heparin fragment (~70 monosaccharide units; ~285 Å long) complexed with AAV2 revealed heparin wound around the shoulders of the 3-fold protrusions, bound by interactions of its sulfate groups with basic arginines and other polar interactions [133]. However, conformational adjustments were very modest and local to the binding site—there was no indication of a capsid-opening conformational change.

Belief in large-scale glycan-induced conformational change persisted due to hints thereof in an independently determined structure of an AAV2-heparin complex at 18 Å resolution [162]. At the low resolutions of both studies, there is increased danger of mis-characterization, so the difference could not be adjudicated immediately. Subsequently, to be more definitive, AAV-DJ [30,34], which shares an AAV2-like HBD, was used for *cryo*-EM of an SOS complex at 4.8 Å resolution [164] and for a complex with a synthetic pentasaccharide heparin analog, fondaparinux at 2.8 Å resolution [160]. The *cryo*-EM maps were much improved, due to chemical homogeneity of the HS analogs and improving EM technology. It was now seen that the AAV structure could make local adaptations to different glycan sequences, but that no large-scale changes were induced by binding. Indeed, evidence was also emerging through competition surface plasmon resonance (SPR) and glycan arrays that the glycan sequence specificity was quite low and that high avidity was achieved with longer heparin oligosaccharides that could bridge between symmetry-related binding sites, combining the affinities thereof [93,165]. The combined evidence implies AAV HSPG attachment is a product of multiple weak-avidity sites on a single capsid. The locations of HSPG attachment can vary between serotypes, and HSPG attachment can be rapidly selected for or against in the laboratory environment.

#### 3.1.2. SIA and GAL

Sialic acid (SIA) was the first virus receptor to be discovered [166,167] and serves as a receptor or attachment factor for many viruses. SIAs are a diverse group of nine-carbon sugars that attach to the end of O-linked (serine or threonine) or N-linked (asparagine) sugar chains. Coronavirus, influenza, and other zoonotic viruses use glycan oligosaccharides terminated in SIA for cellular entry, and SIA may play an important role in crossing species barriers [168]. In parvoviruses, SIA attachment determines MVM tropism and pathogenicity [169] whereas CPV and FPV SIA attachment is not required for infection [170].

O-linked and N-linked SIA have been reported as receptors for several AAVs, although, like HS, SIA should now, more properly, be considered an attachment factor (*vide infra*). Glycan-conjugated SIA is known to interact with AAV1, AAV4, AAV5, and AAV6 [171,172]. AAV4 attaches to α2,3 SIA on O-linked oligosaccharides whereas AAV5 attaches to α2,3 SIA on N-linked oligosaccharides [171]. On the other hand, AAV1 and AAV6 use both α2,3 and α2,6 SIA on N-linked oligosaccharides for cellular attachment [172]. The 3.5 Å X-ray crystallographic structure of SIA-bound AAV5 was examined, and two candidate sites (A and B) were used as a foundation for site-directed mutagenesis [158]. Mutations to residues in the A site were responsible for N-linked SIA attachment. The X-ray crystallography structures of AAV1 and AAV6 revealed additional N-linked SIA attachment areas consisting of six amino acids [159]. Similar to HSPG attachment sites, SIA attachment sites are not conserved and the mutation of residues identified by X-ray crystallography ablate glycan attachment.

AAV9 is unique in that it has a preference for glycans ending in a terminal galactose (GAL) [173]. Mutational studies and computer modeling of docked structures implicate a patch of residues at the base of the 3-fold axis protrusions [174]. This site is distinct from the HS and SIA sites of other AAVs and further emphasized that, among AAVs, divergent strategies have evolved for the attachment to cell surface glycans.

### 3.2. AAV Proteinaceous Receptors

#### 3.2.1. AAVR

A variety of possible co-receptors proteins have been reported over the years. For example, the hepatocyte growth factor receptor (c-MET) and human fibroblast growth factor receptor-1 (FGFR1) were identified as possible protein co-receptors for AAV2 [175,176]. Platelet-derived growth factor receptor (PDGFR) is a candidate receptor for AAV5 [177] while epidermal growth factor receptor (EGFR) was identified as a candidate receptor for AAV6 [178]. A report identifying α_5_β_5_ integrin as an AAV2 co-receptor [179] was contested by another study [180]. Other proposed receptors included integrin α_5_β_1_ [181], LamR for a variety of serotypes [182], and an unidentified 150 kDa glycoprotein [183]. The candidate ~150 kDa protein could be qualitatively observed using virus overlay assays and binding was quantified using cell culture binding assays. Cell-binding could be ablated by trypsinization, with a time-dependent recovery that suggested the involvement of a protein with an eight-hour turnover [183]. The identity of the 150 kDa protein receptor responsible for wtAAV2 binding would remain a mystery for two decades.

Convincing evidence of a cellular entry receptor was eventually found using a high-throughput forward genetic screen to identify conclusively genes involved in AAV transduction [37]. Keys to successful screening were a nearly haploid human cell line (HAP1), a retrovirus gene-trap used to mutagenize most non-essential genes [184], and, because AAV is not cytopathic, methods for iteratively selecting viral resistance based on fluorescence-encoding viral vectors and cell sorting. Then, using an AAV2 vector encoding RFP, repeated fluorescence-activated cell sorting (FACS) cycles were used to select a cell population enriched in AAV-resistant (RFP-negative) mutants. Deep sequencing of cells yielded a list of 46 genes that were mutated with statistically significant frequencies. These could be grouped into genes encoding proteins involved in trans-Golgi network trafficking, heparan sulfate synthesis, a handful of “other” hits and three genes encoding transmembrane proteins. The three transmembrane proteins (KIAA0319L, GPR108, and TM9SF2) were relatively uncharacterized and had not been previously implicated as viral entry factors. The gene candidate with the highest significance (570 independent mutations) was a type I transmembrane protein known as KIAA0319L and was subsequently renamed to AAV Receptor (AAVR).

CRISPR-Cas9 was used to create knockouts (KO) of AAVR in eight diverse human and mouse cell lines and TALENs were used to create AAVR^KO^ mice [37]. The AAVR^KO^ cell lines were resistant to infection with AAV2, even at high doses of 100,000 viral genomes per cell. Infectivity could be restored in AAVR^KO^ cells by expressing AAVR and overexpression of AAVR in cell lines resistant to AAV2 infection rendered the cells permissive to AAV2 infection [37]. AAV2 infection was inhibited via the introduction of soluble AAVR ectodomain or antibodies against AAVR, thereby highlighting the potential importance of access to AAVR on the cell surface. Several other human and simian serotypes (AAV1, 2, 3B, 5, 6, 8 and 9), with preferences for diverse glycan attachment factors, were also unable to infect AAVR^KO^ cells, but could infect cells rescued with AAVR. Similarly, another study found overexpression of AAVR in polarized epithelial cells generates preferentially basolateral localization of AAVR and increased transduction of AAV2 on the basolateral side [185]. In contrast, CRISPR-Cas9 KO of two of the top previously-implicated AAV2 candidate co-receptors (c-MET and FGFR1), in several cell lines, did not decrease transduction efficiency [37]. These results highlighted the importance of AAVR as a primary cell entry receptor for AAV2 and suggested other previously identified AAV2 candidate receptors may play, at most, accessory roles.

**Figure 4:**
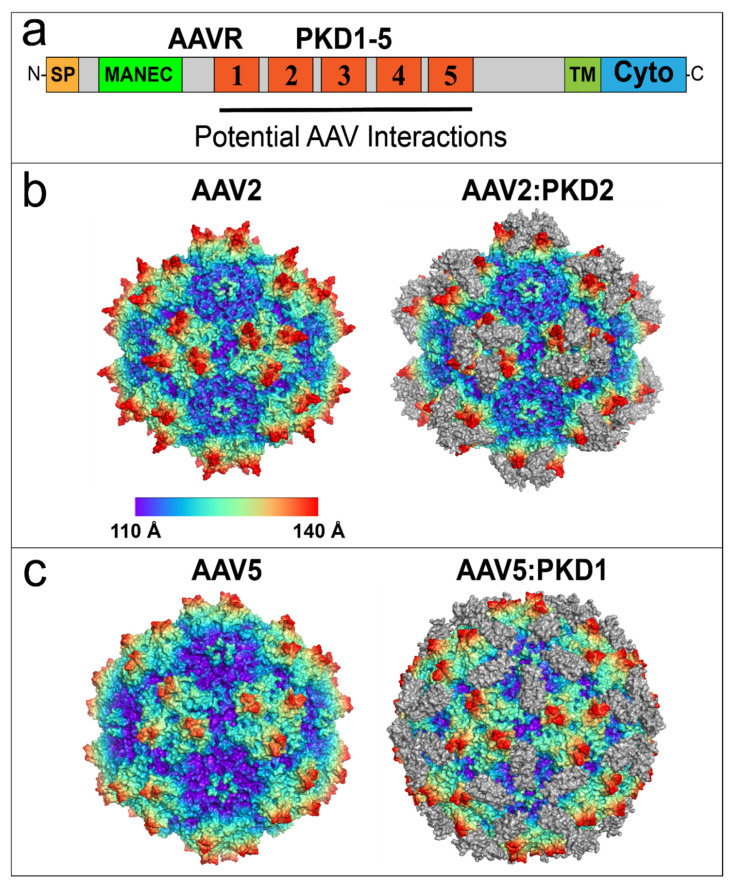
AAV2 and AAV5 bind to distinct AAVR PKD domains. (**a**) AAVR domain structure from N-terminus (N) to C-terminus (C): Signal Peptide (SP), Motif At the N-terminus with Eight Cysteines (MANEC), immunoglobulin-like Polycystic Kidney Disease (PKD) domains 1–5, TransMembrane (TM) helix and Cytoplasmic domain (Cyto). The region containing potential AAV interactions is composed of PKD1-5. (**b**) Native AAV2 60-mer (left) and the AAV2:PKD2 complex (right). Virus models are colored by radial distance from the center of the virion. The PKD2 domain of AAVR is colored in gray. (**c**) Virus model of native AAV5 virion (left) and the AAV5:PKD1 complex (right). Models are colored by radial distance from the center of the virion. The PKD1 domain of AAVR is colored in gray. Structures in (**b**,**c**) were prepared using PyMOL [66].

The dependence of AAV2 on AAVR for infection warranted further investigations into the protein domains controlling infection. AAVR is an N-linked and O-linked glycoprotein that can be subdivided into three regions: an N-terminal motif with eight cysteines (MANEC domain), five immunoglobulin-like polycystic kidney disease (PKD) domains (PKD1-PKD5) and a C-terminal transmembrane region (Figure 4a). AAVR proved to be the same as the previously implicated but unidentified ~150 kDa glycoprotein [183,186], although it became clear that the ~50kDa glycosyl moieties were not required for AAV2 infection [186]. AAVR was found to colocalize with TGN components, as does AAV2 when trafficking from the plasma membrane through endosome compartments to the Golgi [142,186]. Removal of the AAVR C-terminal domain, which contains endosome targeting motifs, prevented rescue of AAVR^KO^ cells and impaired endocytic recycling; resulting in increased AAVR localization to the plasma membrane [186]. The PKD domains of AAVR have immunoglobulin-like (Ig-like) folds that are common in viral receptors [187]. A soluble ecto-domain construct, containing PKD1-5 domains was able to bind AAV2 particles, and a mini-AAVR construct consisting of PKD1-3 together with the transmembrane moiety was sufficient to rescue AAV_KO_ cells [37]. Further analysis with both domain expression constructs and domain deletion mutants that, for AAV1, AAV2 and AAV8, there were strong interactions with PKD2, and subsidiary involvement of PKD1 [186]. By contrast, AAV5 primarily utilizes PKD1, apparently exclusively [186]. The amino acids responsible for AAV-AAVR interactions were soon revealed in a series of structural studies (Table 3; Figure 4b,c) [119,120,188,189]. The footprint of AAV2:PKD2 was found to overlap with a significant fraction (39–56%) of the eighteen residues comprising the previously identified dead zone [118,119,120]. The overlapping footprints of AAVR and antibodies provide additional insights into AAV-antibody neutralizing and cell entry mechanisms.

#### 3.2.2. GPR108 and VP1u

In addition to AAVR-dependent serotypes, at least one primate AAV lineage does not require AAVR for cell transduction and multiple serotypes still exhibit low levels of transduction in the absence of AAVR. Anc80 is predicted (in silico) to be ancestral to AAV2 (Section 2.1) and this lineage is typified by PKD2 binding whereas the lineage leading to AAV5 binds to PKD1. The lineage leading to AAV4 and AAVrh32.33, on the other hand, is AAVR-independent [190]. Anc80 and its descendants also exhibited low levels of AAVR independence whereas AAV5 is solely dependent upon AAVR for cell transduction [190].

**Figure 5:**
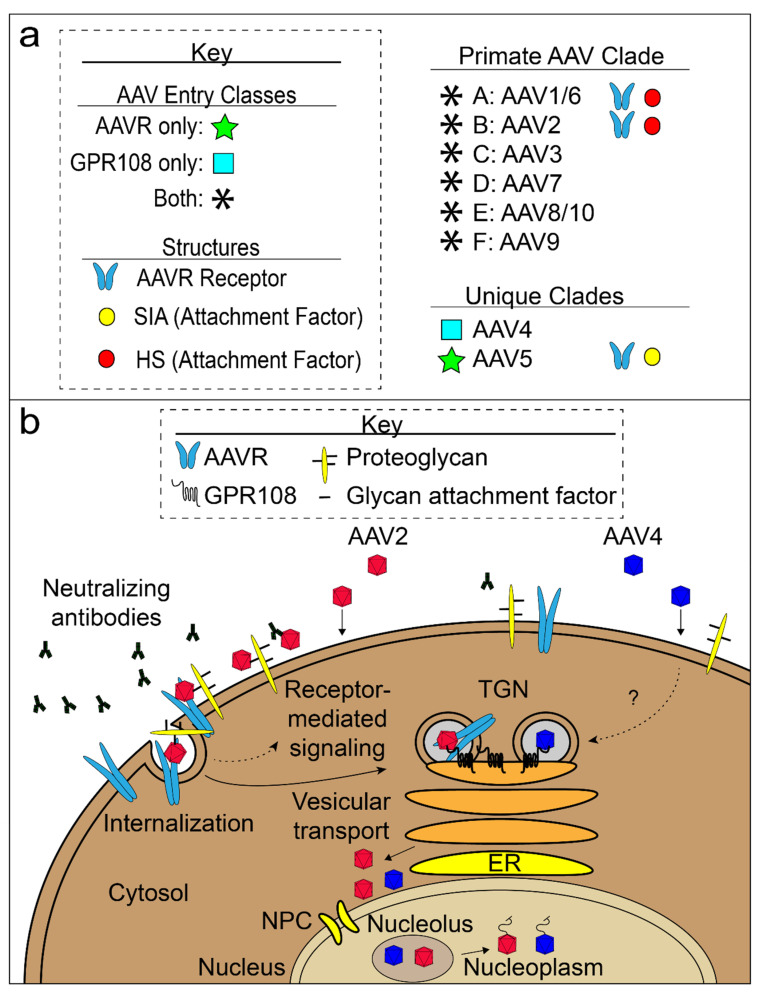
AAV entry model. (**a**) Three known AAV entry classes are shown: AAVR-dependent (green star), GPR108-dependent (light blue box) and both AAVR and GPR108-dependent (black asterisk). Structures for AAV complexes with glycan attachment factors (yellow and red circles) and AAV:AAVR complexes (light blue receptor icon) are known. Appropriate AAV entry class icons are located to the left of AAV serotypes and structural icons are located to the right of AAV serotypes with structural evidence. (**b**) AAV cell attachment, entry, and trafficking to the nucleus of a non-polarized cell. Two AAV classes are shown: AAVR/GPR108-dependent (red; majority of AAVs), represented by AAV2, and GPR108-dependent/AAVR-independent (blue; AAV4 clade). Virions must evade host neutralizing antibodies (black antibody-shaped icons). AAV virions (red or blue) come into contact with the cell surface and attach to the glycan moieties of proteoglycans. Red AAV binds to the AAVR receptor and is internalized and transported through the trans-Golgi network (TGN). Blue, GPR108-dependent, AAV particles enter through a parallel but possibly distinct pathway. Virions accumulate outside of the nucleus before entry and may involve the nuclear pore complex (NPC). AAV gathers in the nucleolus prior to being extruded into the nucleoplasm where the ssDNA genome is released.

Two independent screens validated the Pillay et al. screen hit GPR108 (i.e., Lung Seven Transmembrane Receptor2; LUSTR2) as an important factor for AAV transduction [191,192]. GPR108 is a seven-transmembrane protein with a long N-terminal lumen domain and a short C-terminal domain essential for AAV transduction [191]. Representative members of the Anc80 lineage are dependent on both GPR108 and AAVR whereas the AAV4 clade is mostly dependent just on GPR108 and does not require AAVR for entry [191]. AAV5 does not require GPR108 and the VP1u region of AAV2 can transfer GPR108 dependence to AAV5 [191]. Furthermore, GPR108 expression overlaps with AAVR expression in the TGN [191,192]. Of several possible explanations of the observations, one is that attachment of AAV2-like viruses to HSPG is followed by binding to AAVR, and eventual VP1u extrusion for a GPR108-mediated step in the TGN (Figure 5b) [191].

AAV cellular entry screens are offering insights that are likely relevant to release of VP1u. Both AAVR and GPR108 are predicted transmembrane proteins found in the TGN. Other hits of the forward genetic screens include genes implicated in glycan biosynthesis and TGN functions. One TGN gene high on the list of genes identified by Pillay et al. is ATP2C1. This gene encodes a secretory pathway Ca(2+)-ATPase pump type 1 (SPCA1) important for sequestering calcium ions into the Golgi compartment from the cytosol and also for Golgi ribbon maintenance [193]. AAV conformational changes associated with VP1u extrusion are affected by increased cytosolic calcium levels in ATP2C1 KO cells [194]. The VP1u phospholipase is calcium-dependent [195] and can be induced to extrude by heating from 37 °C to 65 °C [196]. VP1u protease activity that targets disordered proteins has also been detected [197]. Further characterization of genes underlying VP1u-associated effects may provide a roadmap for future mechanistic studies of AAV cellular entry.

## 4. Concluding Remarks

The number of rAAV gene therapy options continues to grow. In addition, advances in structural biology provide easier access to molecular details responsible for cell entry and escape from neutralizing antibodies. Such details are beginning to help investigators design increasingly sophisticated capsid modification schemes to improve rAAV vector properties. Much remains to be learned about the key molecular interactions of capsid with host factors, and one would anticipate continuing feedback into vector design over the coming years. There are also areas in which the salient molecular interactions remain largely uncharacterized, such as cellular immune responses, and so progress in vector delivery, at the moment, involves empirical mitigation strategies [35,198].

Particularly exciting, is the more detailed picture of rAAV cellular entry mechanisms that is now emerging. The discovery of AAVR and the resultant AAV-AAVR structures reveal important capsid surface moieties responsible for cell entry. The AAV4 clade primarily uses AAVR-independent mechanisms for cellular entry, and these insights apply to multiple serotypes, with the possible exception of the AAV5 clade. Further characterization of GPR108-mediated cell entry may provide additional tools for rAAV gene therapy improvement. It is important to emphasize that identification of the most critical host factors for entry is recent, their characterization is ongoing, and exploitation of the emerging understanding for improved vector delivery is only just starting.

That said, our changed perspective on the role of extracellular glycans might be equally important. Designated as primary receptors, there was naturally much attention on understanding the molecular basis of interactions and any specificity thereof that could possibly be exploited for targeting, but we now understand that specificity is less than exquisite [93,165]. Real glycan-dependent tropisms, in vivo, could have a variety of non-receptor-mediated explanations, as are now being uncovered. These can include glycan-dependent sequestration at a target site of interest [199] or rate of blood clearance [121]. Glycan interactions certainly continue to be important in vector optimization [76], but the design considerations may be quite different from modulation of cellular entry. Our newer perspective does not negate the long history of evidence, from in vitro assays, that glycans also mediate attachment to infected cells and thereby affect levels of transduction [36], albeit somewhat less critically than protein receptors [37]. However, now the process should be imagined, as in Figure 5b, with glycan attachment concentrating virus at the cell surface with high-valence modest-specificity interactions, common in many other viral families, improving the efficiency of binding to membrane proteins essential for entry and trafficking [200,201]. In overlapping but different ways, both glycan and protein interactions will be important in the development of more efficient and specifically targeted vectors.

The initial steps leading to AAV cellular entry are clearer, but important details remain unanswered. For example, questions addressing the tendency of AAV to bind AAVR more at the surface or in the endosome and the role of candidate co-receptor interactions with AAV remain unresolved. The subsequent function of AAVR in cellular trafficking and potential roles for AAVR or co-receptors in the steps leading to capsid conformational changes preceding endosomal escape also remain inconclusive. Future studies may accurately resolve these important open questions, in light of our current understanding of AAV:AAVR interactions, with an eye on developing more potent rAAV gene therapy vectors.

## Figures and Tables

**Table 1:** Native AAV structures.

Serotype	Clade	Resolution	Year	Tropism ^1^	Tropism ^2^	Tropism ^3^	Reference	PDBid
AAV1	A	X-ray 2.5 Å	2010	Muscle, CNS, heart	Skin, lung, kidney, cervix, bone	Kidney, skin	Ng et al. [21]	3NG9
AAV2	B	X-ray 3.0 Å	2002	Liver, CNS, muscle	Skin, lung, kidney, cervix, liver, bone	Liver, kidney, cervix, retina, skin	Xie et al. [22]	1LP3
AAV3	C	X-ray 2.6 Å	2010	Muscle, stem cells	Skin, lung, kidney, cervix, liver, bone	Skin	Lerch et al. [23]	3KIC
AAV4	Unique	X-ray 3.2 Å	2006	Eye, CNS	Bone	Not detected	Govindasamy et al. [24]	2G8G
AAV5	Unique	X-ray 3.5 Å	2013	CNS, lung, eye	Not detected	Not detected	Govindasamy et al. [25]	3NTT
AAV6	A	X-ray 3.0 Å	2011	Muscle, CNS, heart, lung	Skin, lung, kidney, cervix, bone	Skin	Xie et al. [26]	4V86
AAV7	D	Cryo-EM 3.0 Å	2021	Muscle, CNS	Not detected	Not detected	Mietzsch et al. [20]	7L5Q
AAV8	E	X-ray 2.6 Å	2007	Liver, muscle, pancreas, CNS	Not detected	Not detected	Nam et al. [27]	2QA0
AAV9	F	X-ray 2.8 Å	2012	Broad distribution	Not detected	Not detected	Dimattia et al. [28]	3UX1
AAVrh.39 (AAV10-like)		Cryo-EM 3.4 Å	2020	Muscle (AAV10)	Not tested	Not tested	Mietzsch et al. [29]	6V1T
AAV11		Cryo-EM 2.9 Å	2021	Unknown	Not tested	Not tested	Mietzsch et al. [20]	7L6F
AAV12		Cryo-EM 2.5 Å	2021	Nasal	Not tested	Not tested	Mietzsch et al. [20]	7L6B
AAV13		Cryo-EM 3.0 Å	2021	Not shown	Not tested	Not tested	Mietzsch et al. [20]	7L6I
AAVDJ		Cryo-EM 4.5 Å	2012	Not shown	Not tested	Liver, kidney, cervix, retina, skin, lung	Lerch et al. [30]	3J1Q
AAVDJ		Cryo-EM 1.6 Å	2020	Not shown	Not tested	Liver, kidney, cervix, retina, skin, lung	Xie et al. [31]	7KFR

^1^ Li and Samulski 2020 [32], Review; ^2^ Ellis et al. 2013 [33], Supplementary Table S2; ^3^ Grimm et al. 2008 [34], Supplementary Table S2.

**Table 2:** Structures of AAV with Attachment Factors.

Serotype	Resolution	Year	Reference
AAV2	Cryo-EM 8.3 Å	2009	O’Donnell et al. [133]
AAV3	X-ray 6.5 Å	2012	Lerch et al. [157]
AAV5	X-ray 3.5 Å	2015	Afione et al. [158]
AAV1	X-ray 3.0 Å	2016	Huang et al. [159]
AAVDJ	Cryo-EM 3.0 Å	2017	Xie et al. [160]

**Table 3:** Structures of AAV complexed with AAVR-receptor fragments.

Serotype	Resolution	Year	Reference	PDBid
AAV1	Cryo-EM 3.3 Å	2019	Zhang et al. [189]	6JCQ
AAV2	Cryo-EM 2.8 Å	2019	Zhang et al. [120]	6IHB
AAV2	Cryo-EM 2.4 Å	2019	Meyer et al. [118]	6NZO
AAV5	Cryo-EM 3.2 Å	2019	Zhang et al. [189]	6JCS
AAV5	Cryo-EM 2.5 Å	2020	Silveria et al. [188]	7KPN

## Supplementary Materials

The following are available online at https://www.mdpi.com/article/10.3390/v13071336/s1. Suplementary Table S1: AAV Mutant Transduction Table. Supplementary Table S2: AAV Tropism Tables.
